# Mutations Affecting Cellular Levels of Cobalamin (Vitamin B_12_) Confer Tolerance to Bactericidal Antibiotics in *Burkholderia cenocepacia*

**DOI:** 10.4014/jmb.2406.06028

**Published:** 2024-07-11

**Authors:** Dongju Lee, Jongwook Park, Heenam Stanley Kim

**Affiliations:** Division of Biosystems and Biomedical Sciences, College of Health Sciences, Korea University, Seoul 02841, Republic of Korea

**Keywords:** *Burkholderia cepacia* complex (Bcc), *Burkholderia cenocepacia*, pneumonia, cobalamin, vitamin B_12_, tobramycin

## Abstract

The *Burkholderia cepacia* complex (Bcc) consists of opportunistic pathogens known to cause pneumonia in immunocompromised individuals, especially those with cystic fibrosis. Treating Bcc pneumonia is challenging due to the pathogens' high multidrug resistance. Therefore, inhalation therapy with tobramycin powder, which can achieve high antibiotic concentrations in the lungs, is a promising treatment option. In this study, we investigated potential mechanisms that could compromise the effectiveness of tobramycin therapy. By selecting for *B. cenocepacia* survivors against tobramycin, we identified three spontaneous mutations that disrupt a gene encoding a key enzyme in the biosynthesis of cobalamin (Vitamin B_12_). This disruption may affect the production of succinyl-CoA by methylmalonyl-CoA mutase, which requires adenosylcobalamin as a cofactor. The depletion of cellular succinyl-CoA may impact the tricarboxylic acid (TCA) cycle, which becomes metabolically overloaded upon exposure to tobramycin. Consequently, the mutants exhibited significantly reduced reactive oxygen species (ROS) production. Both the wild-type and mutants showed tolerance to tobramycin and various other bactericidal antibiotics under microaerobic conditions. This suggests that compromised ROS-mediated killing, due to the impacted TCA cycle, underlies the mutants’ tolerance to bactericidal antibiotics. The importance of ROS-mediated killing and the potential emergence of mutants that evade it through the depletion of cobalamin (Vitamin B_12_) provide valuable insights for developing strategies to enhance antibiotic treatments of Bcc pneumonia.

## Introduction

The *Burkholderia cepacia* complex (Bcc) consists of at least 24 closely related species that are opportunistic human pathogens [1-3]. Bcc species often cause pneumonia in immunocompromised individuals with underlying lung diseases, particularly cystic fibrosis (CF) patients [4-6]. The clinical manifestations of Bcc pneumonia in CF patients are often more severe and progress more rapidly than those caused by *Pseudomonas aeruginosa* [4-6]. Besides causing lung inflammation, Bcc species can cross the epithelial barrier and enter the systemic circulation, leading to bacteremia [7]. *B. cenocepacia*, in particular, is associated with a mortality rate up to five times higher than that of other Bcc species and constitutes an exclusion criterion for lung transplantation due to the high risk of death [8-10]. Unfortunately, 85–97% of lung infections caused by Bcc worldwide are by *B. cenocepacia* and *B. multivorans* [8-10], with 94% and 50% of the respective infections being chronic despite aggressive antibiotic therapies [11].

Bcc pneumonia is challenging to manage due to the inherent resistance of the pathogens to many commonly used antibiotics, including carboxypenicillins, first- and second-generation cephalosporins, polymyxins, and aminoglycosides [12, 13]. While there is still no consensus or guideline on the therapeutic regimens for people with CF infected by Bcc [14], the management of Bcc infections typically involves aggressive antibiotic regimens, including intravenous, inhaled, and oral antibiotics [15-17]. Tobramycin, an aminoglycoside, has been commonly used to treat pneumonia caused by Bcc or *P. aeruginosa* [18, 19]. Administered in an inhaled form, tobramycin achieves high lung concentrations, potentially overcoming Bcc's intrinsic resistance to the antibiotic [20, 21]. In fact, a regimen of intravenous and inhaled tobramycin, ceftazidime, trimethoprim/sulfamethoxazole, and azithromycin have been shown to clear Bcc from sputum cultures and stabilize CF lung disease after one year [16]. In another study, successful eradication of new Bcc isolates in two children with CF using a combination of intravenous tobramycin, ceftazidime, and temocillin, followed by three months of inhaled tobramycin, has been reported [15]. However, a study using inhaled tobramycin did not demonstrate statistically significant improvements in lung function, although reductions in sputum bacterial density and inflammation markers, such as interleukin-8 (IL-8) levels, were observed [20]. Additionally, a combination of inhaled amiloride and tobramycin eradicated initial colonization in 3 out of 4 patients but was unsuccessful in chronic infections [22, 23].

In this study, we investigated potential mechanisms by which tobramycin becomes less effective in treating Bcc pneumonia. By focusing on *B. cenocepacia*, a crucial species for Bcc’s pathogenicity, we discovered that mutations in a gene essential for the biosynthesis of cobalamin (Vitamin B_12_) confer tolerance to tobramycin and various other bactericidal antibiotics often used in current antibiotic regimens. This novel mechanism operates by depleting cobalamin (Vitamin B_12_) levels, which ultimately impacts tricarboxylic acid (TCA) cycle-dependent reactive oxygen species (ROS)-mediated killing, a common endpoint action of bactericidal antibiotics [24-27]. These findings provide valuable insights for developing more effective therapeutic strategies against Bcc pneumonia.

## Materials and Methods

### Bacterial Strains and Growth Conditions

*Escherichia coli* strains were cultured in Luria Bertani (LB) medium (Becton, Dickinson and company, USA), and *B. cenocepacia* strains were cultured in Iso-sensitest medium (Oxoid, United Kingdom) at 37°C [28]. Kanamycin was used at 50 μg/ml for *B. cenocepacia*, and tetracycline was used at 10 μg/ml for *E. coli* and 150 μg/ml for *B. cenocepacia* with a cloning vector, pRK415 [29].

### Selection for Surviving Mutants against Tobramycin

*B. cenocepacia* strain J2315 was grown on Iso-sensitest agar at 37°C for 2 days. A single colony was inoculated into 2 ml of Iso-sensitest broth and grown overnight at 37°C with shaking at 250 rpm. The overnight culture was washed with fresh Iso-sensitest broth and diluted to approximately 10^7^ CFU/ml. Then, 100 μl of cell suspension was spread on Iso-sensitest agar plates containing 400 μg/ml tobramycin, and the plates were incubated at 37°C for 48 h or until colonies were visible. As a result, we isolated 11 colonies that survived the lethal level of tobramycin.

### Mapping Mutations Conferring Reduced Susceptibility to Tobramycin

To identify mutated genes associated with reduced susceptibility to tobramycin, genomic DNA was purified from 2 of the 11 tobramycin-resistant isolates using a Wizard Genomic DNA Purification Kit (Promega, USA) and sequenced on a HiSeq 2000 platform (Illumina, USA) at Macrogen Inc. (Republic of Korea). A DNA library of genomic DNA fragments was prepared using the TruSeq Nano DNA Kit (Illumina, USA), following Illumina protocols. The library’s quality was assessed using the Agilent 2100 Bioanalyzer (Agilent Technologies, USA), and sequencing was performed as paired-end 101-bp reads on a HiSeq 2000 (Illumina) at Macrogen Inc. Data analysis was conducted using the CLC Genomics Workbench software (Qiagen, USA). In brief, raw sequence data underwent paired-end trimming, and the resulting reads were mapped to the *B. cenocepacia* J2315 reference genome (accession numbers: AM747720.1 (chromosome 1), AM747721.1 (chromosome 2), AM747722.1 (chromosome 3), and AM747723.1 (plasmid pBCJ2315)). As a result, we identified a mutation in the gene BCAL2923 in one of the genomes; however, we did not identify a mutation in the other genome.

To determine if the 9 isolates that we did not sequence have mutations in BCAL2923, genomic DNA was purified using a Wizard Genomic DNA purification kit (Promega) and used as the template for PCR amplification. Amplicons spanning the coding region of the BCAL2923 gene and short flanking regions (162 bp upstream of the start codon and 94 bp downstream of the stop codon) were generated by PCR using primers BCAL2923-F (5’-GGCATGTTTCGCAATCTGTA-3’) and BCAL2923-R (5’-ACCCGCTCGATGACTGAAT-3’). The PCR reaction was performed in a 50 μl reaction mixture containing 1U of KOD FX Neo polymerase (Toyobo, Japan), 25 μl of 2X KOD FX Neo buffer, 10 μl of dNTP mix (2 mM each), 0.3 mM of each primer, and 100 ng of template genomic DNA. The PCR cycling conditions were as follows: initial denaturation at 94°C for 2 min, followed by 35 cycles of denaturation at 98°C for 10 s, annealing at 60°C for 30 s, and extension at 68°C for 40 s, with a final extension step at 72°C for 7 min using the C1000 Thermal Cycler (Bio-Rad Laboratories, USA). The PCR products were purified using a PCR Purification Kit (DyneBio Inc., Republic of Korea) and sequenced bidirectionally using the primers BCAL2923-F and BCAL2923-R on a 3730 DNA analyzer (Applied Biosystems, USA). From this PCR screening, we identified 2 additional isolates with a mutation in the BCAL2923 gene.

### Cloning of the BCAL2923 Gene and Transformation of *B. cenocepacia* Strains with Plasmids

To obtain the BCAL2923 gene, the entire putative operon, including three genes (BCAL2925, BCAL2924, and BCAL2923), was PCR-amplified using KOD FX Neo polymerase (Toyobo) and genomic DNAs extracted from *B. cenocepacia* strains. The primers used were BCAL2925-KF (5’-ATATATGGTACCGATGGTCTGCTCGATTGTCC-3’) and BCAL2923-XR (5’-ATATATTCTAGAACCCGCTCGATGACTGAAT-3’), incorporating the KpnI (NEB; USA) and XbaI (NEB) recognition sites (underlined), respectively. The amplification spanned from 378 bp upstream of the start codon of the BCAL2925 gene to 94 bp downstream of the stop codon of the BCAL2923 gene. The PCR products were digested with KpnI and XbaI and then ligated with the broad-host-range vector pRK415 [29], which had been previously digested with KpnI and XbaI. Following this, the ligation mixture was used to transform *E. coli* DH5α by a traditional transformation method [30]. The transformed *E. coli* strains were cultured on LB agar plates containing 10 μg/ml tetracycline and 500 μg/ml X-gal. The final plasmid construct was then extracted from the chosen *E. coli* strain and subsequently used to transform *E. coli* strain S17-1 [31]. The transformed *E. coli* strains were grown on LB agar plates containing 10 μg/ml tetracycline. S17-1 strains harboring plasmids underwent conjugation [32] with *B. cenocepacia* strains on Iso-sensitest agar plates supplemented with 150 μg/ml tetracycline and 50 μg/ml kanamycin. The plates were incubated at 37°C for 2 or 3 days to select transconjugants. Successful conjugation was confirmed by purifying the plasmid and analyzing its characteristic restriction patterns.

### Determination of Minimum Inhibitory Concentration (MIC)s for Antibiotics

MICs for antibiotics were determined using the agar dilution method [33]. A single colony of each *B. cenocepacia* strain grown on Iso-sensitest agar was inoculated in 2 ml of Iso-sensitest broth. The inoculums were incubated overnight at 37°C with shaking at 250 rpm. Overnight cultures were diluted with fresh Iso-sensitest broth to 1 × 10^7^ CFU/ml. 1 μl of diluted bacterial suspension (approximately 1 × 10^4^ bacterial cells) was dropped onto Iso-sensitest agar plates containing antibiotics at various concentrations using a multichannel micropipette. The plates were incubated at 37°C for 18 h, and then the MICs were determined as the lowest concentration of antibiotics at which no visible colonies were observed. The concentration of 1 × 10^7^ CFU/ml was confirmed by spreading 100 μl of serial dilutions from bacterial suspensions onto Iso-sensitest agar plates, followed by incubation at 37°C for 48 h and subsequent enumeration of visible colonies. Each experiment was conducted at least three times.

### Quantification of ROS in a Bacterial Culture

Single colonies of *B. cenocepacia* strains J2315, J-M1, J-M2, and J-M3 were inoculated into 2 ml of Iso-sensitest broth and incubated overnight at 37°C with shaking at 250 ×*g*. The overnight cultures were used to inoculate 20 ml of fresh Iso-sensitest broth at a ratio of 1:100 and were further incubated at 37°C with shaking at 250 rpm. Upon reaching an OD_600_ of 1.0, the cultures were divided into three separate test tubes, each containing 3 ml. These aliquots were treated with tobramycin at concentrations of 0, 600, and 900 μg/ml, respectively, and then incubated at 37°C with shaking at 250 rpm for 4 h. After incubation, 600 μl from each sample was withdrawn, and the cells were collected by centrifugation at 4°C and 5000 g for 4 min. The resulting cell pellets were resuspended in 297 μl of phosphate-buffered saline (PBS) and mixed with 3 μl of 1 mM 5-(and-6)-chloromethyl-2’, 7’-dichlorofluorescin diacetate (CM-H_2_DCFDA) (Molecular Probes, Eugene, OR, USA) solution in DMSO. Following a 1-hour incubation protected from light, cells were washed with PBS and dispensed into two wells of a 96-well plate, with each well containing 100 μl of the sample. Intracellular ROS content was quantified using a SpectraMax M2 microplate reader (Molecular Devices, USA) at excitation and emission wavelengths of 485 and 535 nm, respectively. Additionally, the cells were placed on slides with cover glasses and photographed using a fluorescence microscope (Axiovert, Zeiss, Germany). To assess the concentration of the cultures post-antibiotic treatment, 100 μl of serial dilutions was plated onto Iso-sensitest agar plates, and visible colonies were enumerated after incubation.

### Antibiotic Disc Assays under Aerobic and Microaerobic Conditions

To perform antibiotic disc assays, 200 μl of overnight cultures of *B. cenocepacia* strains was mixed with 3 ml of 0.7% agar and gently overlaid onto Iso-sensitest agar plates. Paper discs were soaked with the following antibiotic solutions: 10 μl of tobramycin (TOB; 50 mg/ml), 20 μl of kanamycin (KAN; 50 mg/ml), 10 μl of ceftazidime (CAZ; 20 mg/ml), 10 μl of meropenem (MER; 5 mg/ml), 3 μl of ciprofloxacin (CIP; 10 mg/ml), 5 μl of chloramphenicol (CHL; 24 mg/ml), and 20 μl of trimethoprim (TMP; 24 mg/ml). The antibiotic discs were air-dried and then placed on the plates. The plates were incubated at 37°C under either aerobic conditions or microaerobic conditions (using the GasPak EZ CampyPouch system; BD, USA) until visible inhibition zones appeared.

## Results

### Mutations in the BCAL2923 Gene Conferred Decreased Susceptibility to Tobramycin in *B. cenocepacia*

**Fig. 1:**
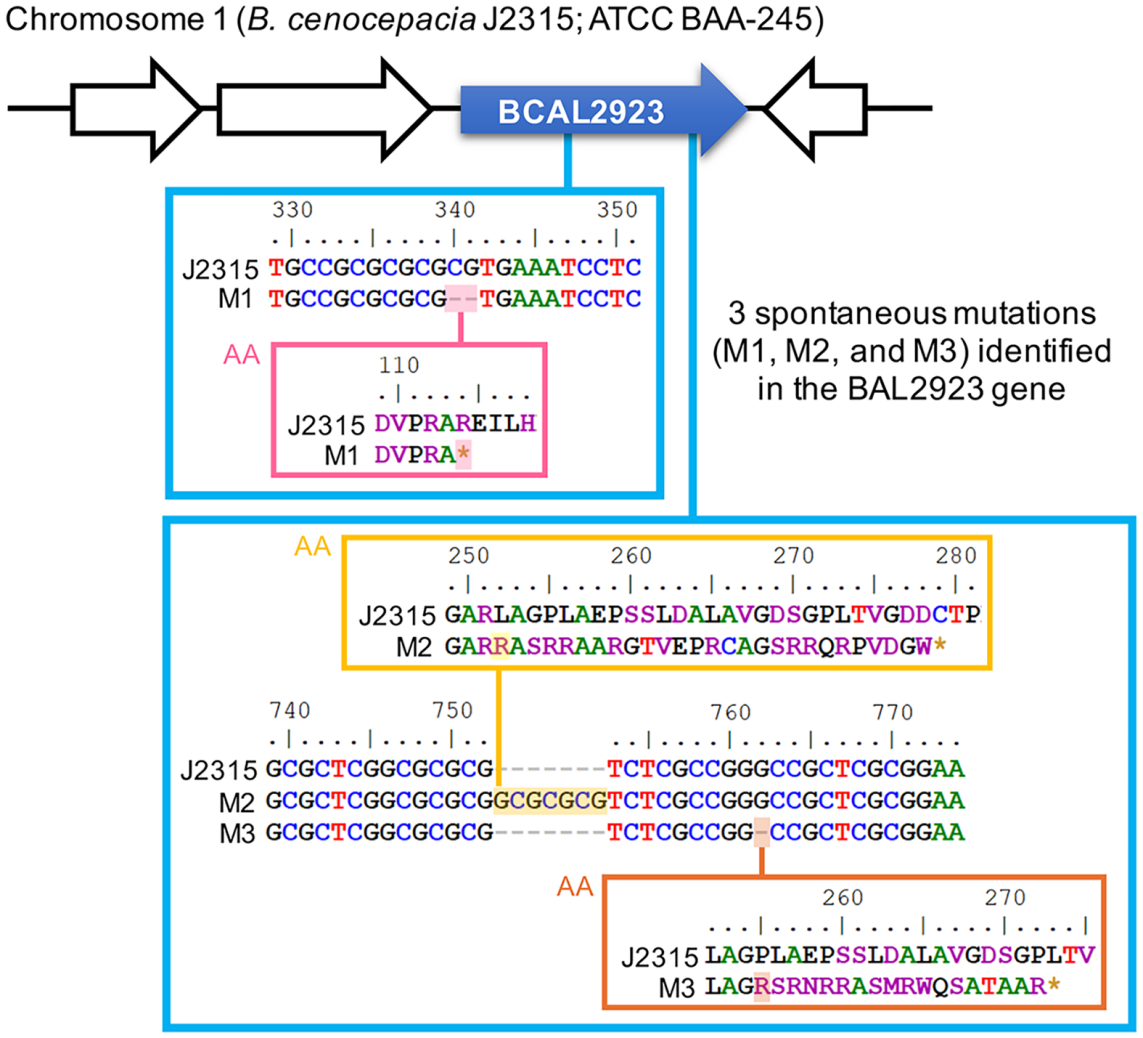
Mutations in the BCAL2923 gene conferred decreased susceptibility to tobramycin in *B. cenocepacia*. A mutation (M1) and two closely located mutations (M2 and M3) in the gene are displayed separately in blue boxes, connected to their approximate locations in the gene. Mutations highlighted in colors are shown at both the nucleotide level and the amino acid (AA) level, displayed in separate boxes.

In exploring potential mechanisms that may impact the effectiveness of tobramycin therapy for Bcc pneumonia, we focused on *B. cenocepacia*, a crucial species of Bcc [8-10]. *B. cenocepacia* strain J2315, originally isolated from a CF patient in the United Kingdom [34], was subjected to antibiotic selection using a lethal concentration of tobramycin at 400 μg/ml. Subsequently, three mutations were identified in three different surviving colonies through whole genome sequencing and PCR screening. All these mutations were in the BCAL2923 gene, which encodes adenosylcobinamide-phosphate synthase, a CobD/CbiB family protein predicted to be involved in the cobalamin (Vitamin B_12_) biosynthesis pathway [35, 36] (Fig. 1). The mutations were a two-base pair deletion (M1), a seven-base pair insertion (M2), and a one-base pair deletion (M3) (Fig. 1). Each of these mutations severely disrupted the gene and consequently the enzyme, causing a frameshift followed by early translation termination.

**Fig. 2:**
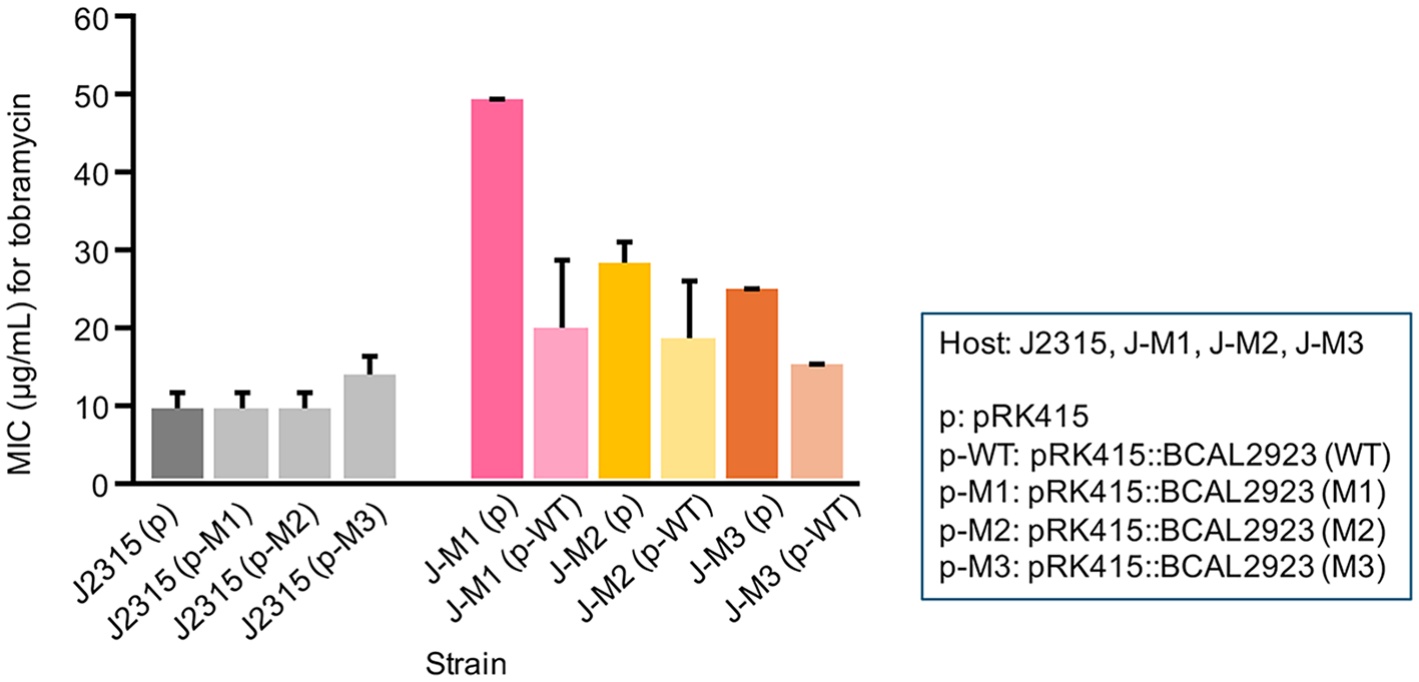
The mutations in the BCAL2923 gene are the sole determinant for the decreased susceptibility to tobramycin. The MICs for tobramycin for each strain carrying different plasmids are shown (mean ± S.D., n ≥ 3).

To confirm that a mutation in the BCAL2923 gene was solely responsible for the survival of the *B. cenocepacia* strain against tobramycin, we conducted complementation analyses. First, the wild-type and the mutant genes were cloned into a vector (*i.e.*, pRK415), and the plasmids were introduced into *B. cenocepacia* J2315. Conversely, a plasmid with the wild-type gene was introduced into each mutant. Introducing the mutant genes into strain J2315 did not significantly alter the MICs of strain J2315 for tobramycin (Fig. 2). However, introducing the wild-type gene into each mutant significantly decreased the MICs for tobramycin in the mutants (Fig. 2). These results confirmed that the mutations were the sole determinant of the decreased susceptibility to tobramycin and also indicated that these mutations are deleterious and recessive.

### Mutations in the BCAL2923 Gene conferred Decreased Susceptibility to Various Antibiotics besides Tobramycin

**Fig. 3:**
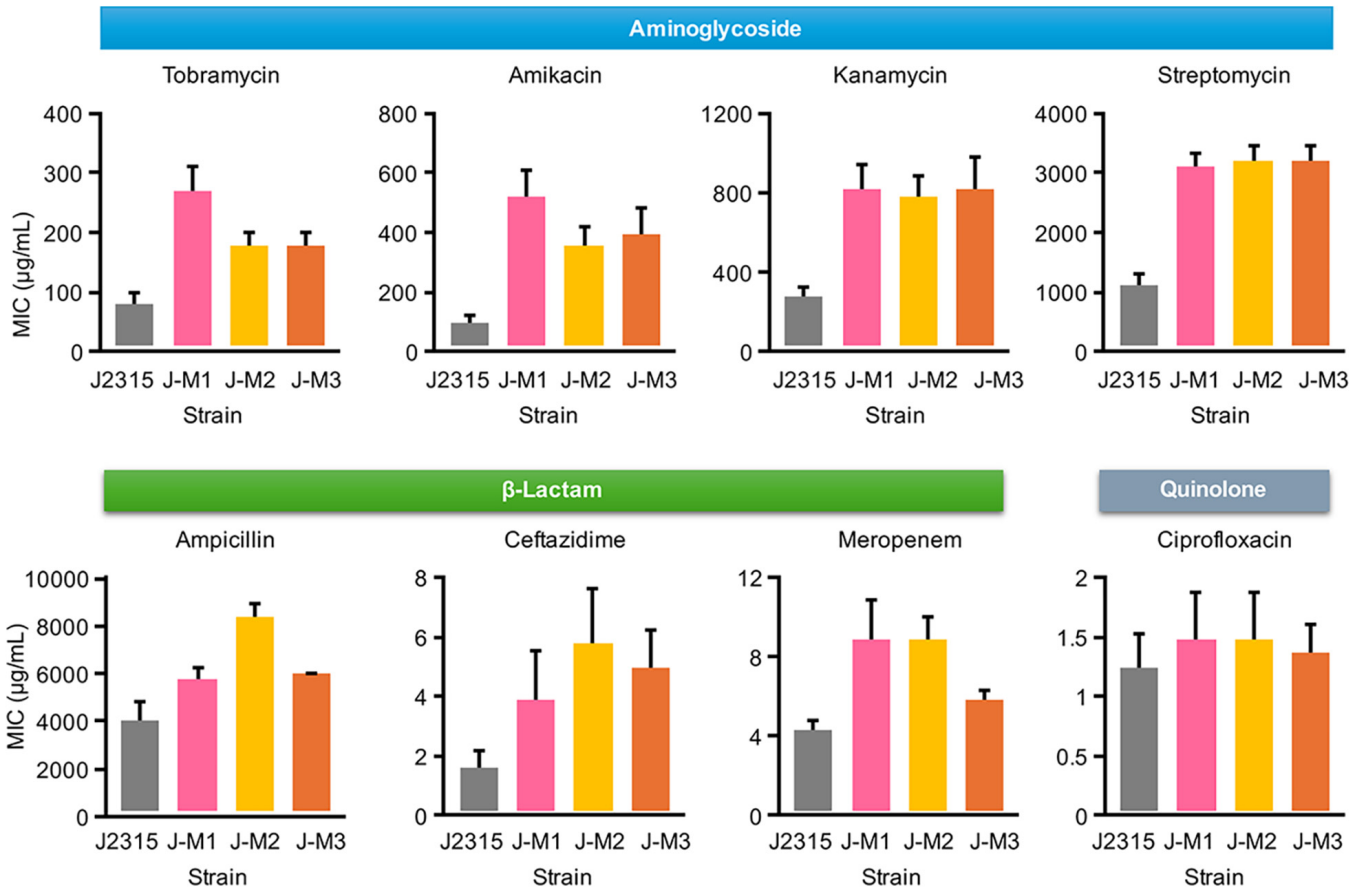
The BCAL2923 mutants exhibit decreased susceptibility to various aminoglycosides, β-lactams, and a quinolone ciprofloxacin. Bar graphs depict the MICs for these antibiotics (mean ± S.D., n ≥ 3).

The mutants exhibited reduced susceptibility not only to tobramycin but also to other aminoglycosides, including amikacin, kanamycin, and streptomycin, with at least a threefold increase in MICs (Fig. 3). Notably, the mutants also exhibited reduced susceptibility to another group of bactericidal antibiotics, β-lactams, including ampicillin, ceftazidime, and meropenem, with at least a twofold increase in MICs (Fig. 3). Additionally, they showed reduced susceptibility to the quinolone ciprofloxacin, although the MIC increase for ciprofloxacin was not as dramatic as for β-lactams (Fig. 3). These results are intriguing because aminoglycosides, β-lactams, and quinolones have different primary cellular target processes: translation, cell wall synthesis, and DNA replication, respectively [25]. Therefore, these results suggest that a tolerance mechanism contributes to reduced susceptibility to tobramycin and various bactericidal antibiotics, rather than a resistance mechanism that would be specific to tobramycin [37, 38]. By contrast, MICs for bacteriostatic antibiotics, such as chloramphenicol and trimethoprim, did not significantly increase (data not shown).

### The Mutations in the BCAL2923 Gene Resulted in Decreased ROS Production upon Exposure to Tobramycin

As the mutations appeared to impact the TCA cycle by depleting cellular levels of succinyl-CoA, we hypothesized that this might impair the production of ROS upon exposure to tobramycin. Studies have suggested that bactericidal antibiotics, despite having different initial interactions with their primary targets, ultimately impact the TCA cycle [24-27]. This leads to the production of ROS, which damage DNA, lipids, and proteins, ultimately resulting in cell death [24-27].

**Fig. 4:**
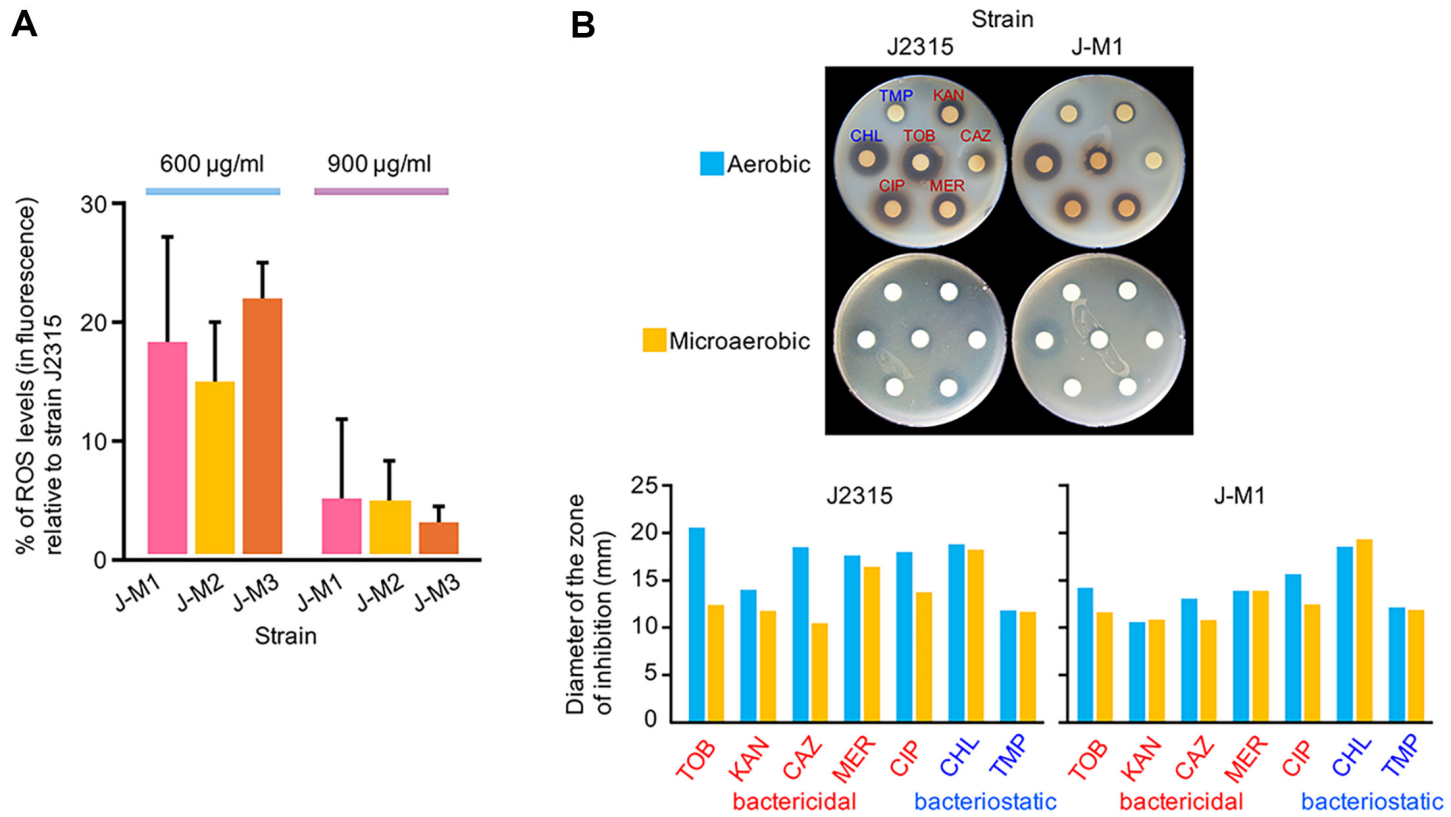
The BCAL2923 mutants produce reduced levels of ROS. **A.** Intracellular levels of ROS production upon exposure to tobramycin. ROS levels in each strain were quantified after exposure to tobramycin at concentrations of 600 μg/ml or 900 μg/ml, normalized to cell numbers, and converted into percentages of the value of strain J2315 (mean ± S.D., n ≥ 3). **B.** Antibiotic susceptibility assays conducted under aerobic and microaerobic conditions. The zones of inhibition of strains J2315 and J-M1 against tobramycin (TOB), kanamycin (KAN), ceftazidime (CAZ), meropenem (MER), ciprofloxacin (CIP), chloramphenicol (CHL), and trimethoprim (TMP) were evaluated using paper discs under both conditions. The actual assay plates are shown at the top, with their quantified data displayed in the bar graphs below.

To verify this hypothesis, ROS production in the mutants was measured upon exposure to tobramycin. The results demonstrated that the mutants displayed a considerable reduction in ROS production, approximately 15 to 23% of J2315's levels, upon exposure to 600 μg/ml of tobramycin (Fig. 4A). When exposed to 900 μg/ml of tobramycin, the mutants produced even lower levels of ROS, ranging from approximately 2 to 5% of J2315's levels (Fig. 4A).

To evaluate the significance of ROS in the bactericidal effect of tobramycin in *B. cenocepacia*, the wild-type strain J2315 and a mutant, J-M1, were exposed to tobramycin and additional antibiotics under normal (aerobic) conditions or microaerobic conditions, where ROS production would be affected. Notably, the diameters of the zones of inhibition formed around the paper discs with bactericidal antibiotics, such as tobramycin, kanamycin, ceftazidime, meropenem, and ciprofloxacin, were reduced under microaerobic conditions compared to aerobic conditions in J2315 (Fig. 4B). Similar overall patterns were observed with J-M1, although they were not as clear as in J2315 due to already reduced susceptibility to the antibiotics (Fig. 4B). By contrast, the diameters of the zones of inhibition around bacteriostatic antibiotics, such as chloramphenicol and trimethoprim, did not significantly reduce under microaerobic conditions in either strain (Fig. 4B). These results demonstrated that ROS-mediated killing is significant in the bactericidal activity of tobramycin and other bactericidal antibiotics, whereas it is not in bacteriostatic antibiotics.

## Discussion

**Fig. 5:**
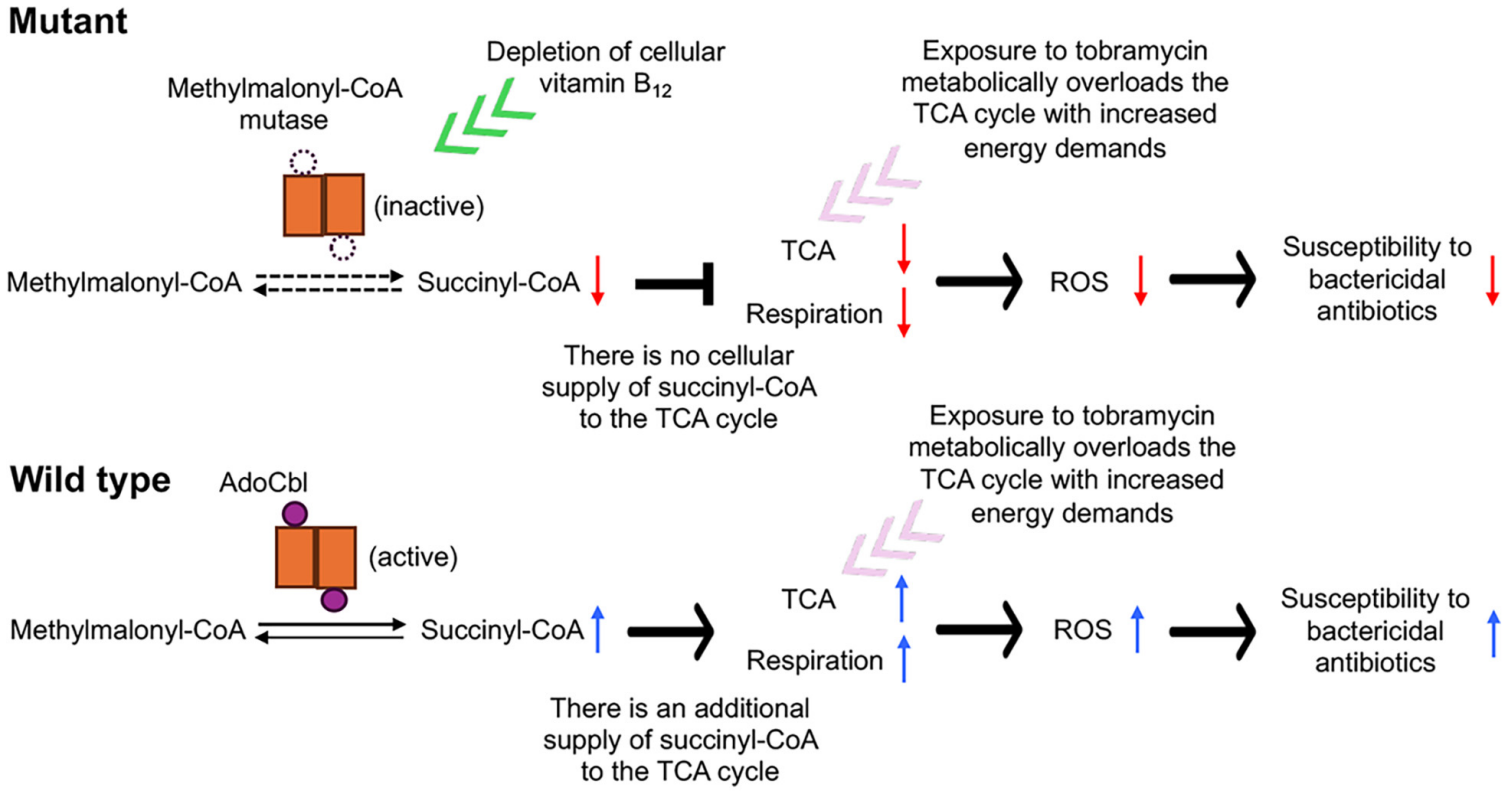
Proposed model for tolerance to tobramycin and other bactericidal antibiotics caused by BCAL2923 mutations in *B. cenocepacia*. Mutations in the BCAL2923 gene lead to a depletion of cellular cobalamin (Vitamin B_12_), affecting the activity of methylmalonyl-CoA mutase, which requires adenosylcobalamin (AdoCbl) as a cofactor. During exposure to bactericidal antibiotics such as tobramycin, the tricarboxylic acid (TCA) cycle becomes metabolically overloaded due to increased energetic demands. Without an additional supply of succinyl-CoA, due to inactive methylmalonyl-CoA mutase, the activities of both the TCA cycle and respiration are impaired, resulting in reduced ROS generation and, consequently, reduced susceptibility to bactericidal antibiotics. In contrast, the wild-type strain, with active methylmalonyl- CoA mutase, produces normal levels of ROS, leading to increased susceptibility to the antibiotics.

By combining the experimental data on mutations in the BCAL2923 gene in *B. cenocepacia* with the widely accepted mechanism of action of bactericidal antibiotics, which involves TCA cycle-dependent ROS-mediated killing [24-27], we propose that cobalamin (vitamin B_12_) depletion leads to reduced efficacy of ROS-mediated killing. This reduction results in the mutants’ tolerance to tobramycin and other bactericidal antibiotics (Fig. 5). This antibiotic tolerance is triggered by mutations disrupting adenosylcobinamide-phosphate synthase (Fig. 1), a key enzyme in the cobalamin (Vitamin B_12_) biosynthesis pathway [35, 36]. This disruption may affect the production of succinyl-CoA by methylmalonyl-CoA mutase, which requires adenosylcobalamin as a cofactor [39]. The depletion of cellular succinyl-CoA may impact the TCA cycle, which becomes metabolically overloaded with increased energetic demands upon exposure to tobramycin [40]. Consequently, the mutants exhibited significantly reduced ROS production, leading to tolerance to tobramycin and other bactericidal antibiotics often used in current antibiotic regimens (Figs. 4 and 5).

Similar to how the depletion of a TCA cycle intermediate succinyl-CoA impacts the TCA cycle and leads to reduced susceptibility to tobramycin in *B. cenocepacia* (Fig. 5), there has been a report showing that glyoxylate has a similar effect by diverting acetyl-CoA through the glyoxylate shunt in *P. aeruginosa* [41]. Conversely, fumarate potentiated tobramycin efficacy by activating the TCA cycle [41]. These observations support our findings that depleted cobalamin (Vitamin B_12_), and consequently most likely also cellular succinyl-CoA, which impact TCA cycle activity, is crucial for susceptibility to tobramycin and many bactericidal antibiotics, including aminoglycosides, β-lactams, and quinolones (Figs. 3, 4, and 5).

Consistent with our data showing that *B. cenocepacia* strains had reduced susceptibility to tobramycin and other bactericidal antibiotics under microaerobic conditions (Fig. 4B), a study reported that clinical isolates of various bacterial genera exhibited increased MICs for aminoglycosides, such as amikacin and tobramycin, under anaerobic conditions [42]. Notably, pathogens from CF patients also showed significant increases in MICs for tobramycin under anaerobic conditions [43, 44]. These findings consistently indicate that ROS production is crucial for the efficacy of bactericidal antibiotics in these pathogens.

In this study, we discovered that, upon exposure to tobramycin, *B. cenocepacia* can acquire a mutation in a gene that leads to the depletion of cobalamin (Vitamin B_12_) and thereby succinyl-CoA, ultimately inducing tolerance to tobramycin and other bactericidal antibiotics (Fig. 5). This novel pathway to antibiotic tolerance provides valuable insights for developing more effective strategies, such as incorporating cobalamin (Vitamin B_12_) supplementation, to potentiate the efficacy of antibiotic treatment for Bcc pneumonia, which is becoming increasingly difficult due to the ever-developing multidrug resistance in Bcc.
